# Supplementary material for: Pause sequences facilitate entry into long-lived paused states by reducing RNA polymerase transcription rates
Source: Nat Commun. 2018 Jul 26;9:2930. doi: 10.1038/s41467-018-05344-9 (PMC6062546; doi:10.1038/s41467-018-05344-9)
Supplement: Supplementary file 1 — Supplementary Information [file 41467_2018_5344_MOESM1_ESM.pdf]

SUPPLEMENTARY INFORMATION

Pause Sequences Facilitate Entry into Long-lived Paused States by Reducing RNA Polymerase Transcription Rates

Gabizon & Lee et al.

Supplementary figures……………………………………2
Supplementary tables…………………………………...9
Supplementary methods...13
References………………………………………………..25

Supplementary Figures:

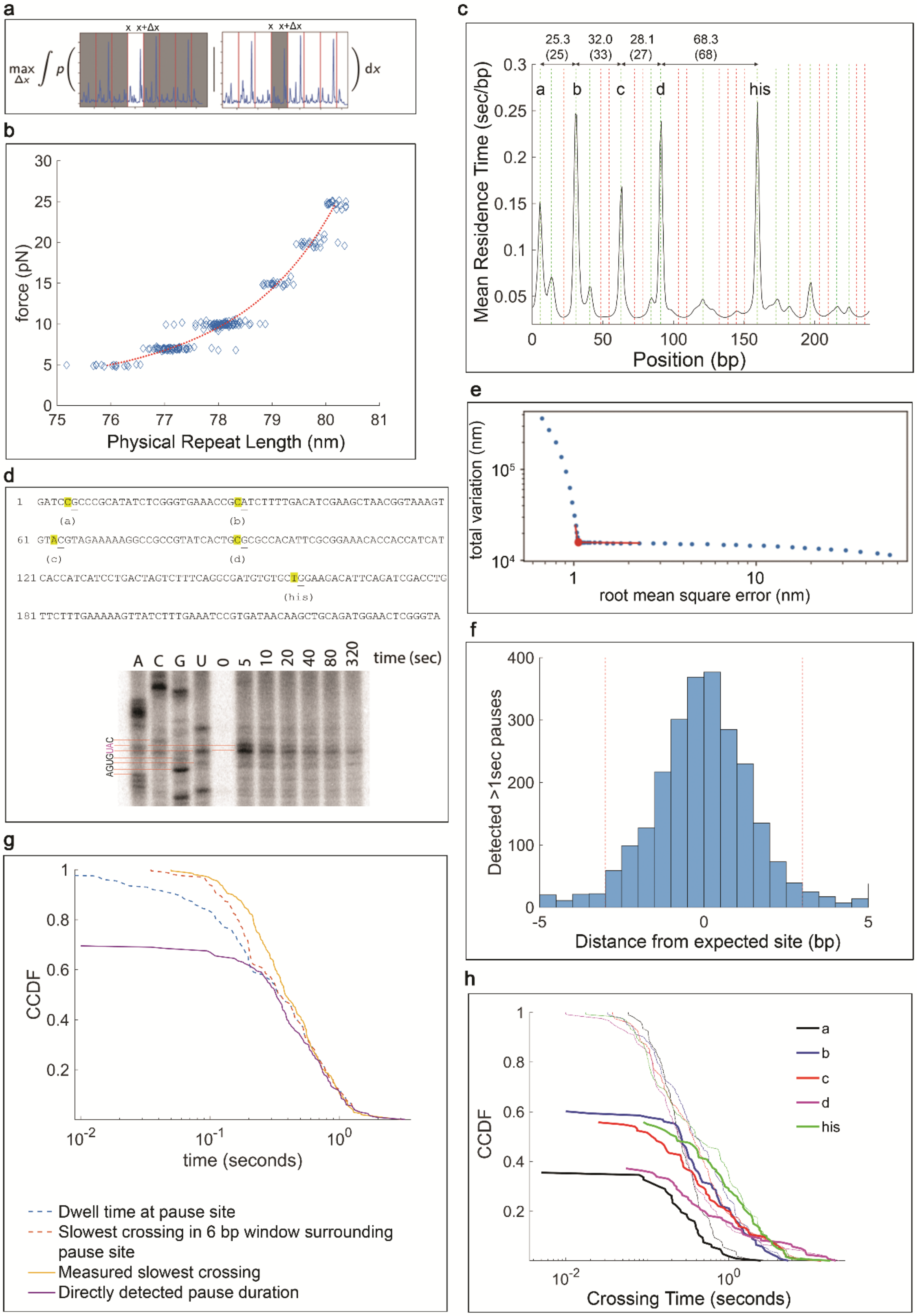

Supplementary Figure 1: Initial alignment and calculation of crossing time distributions
a) Calculation of similarity score.
b) Repeat lengths vs. force for all aligned traces (blue diamonds, N = 251). Red line: Worm-like chain model fitting for the average lengths obtained at every force ($R^2 = 0.999$).
c) Residence time histogram at all conditions, excluding RNase samples. Calculated distance between the main peaks are shown above the arrows (top value: calculated value; value in parenthesis: expected according to Herbert et al.). Green lines: positions of pause sites; red lines: positions of reference (non-pause) sites.
d) Top: sequence of the repeat. The underlined base denotes the +1 base on the non-template strand; the yellow color indicates the 3' end of the RNA at the pause site according to Herbert et al. Bottom: bulk transcription experiment surrounding the area containing pause 'c'. The main pause site is 1 bp upstream of the previously published site. An RNA ladder was generated by performing the reaction in the presence of 3' deoxy NTP's.
e) L-curve for picking the value of the regularizer for the 10 pN assiting force dataset. Each blue dot corresponds to an individual value of λ (taken at a constant ratio, 2^(1/3), of each other). The two red segments are tangents to the L-curve at its inflection; their intersection is marked by a red dot; the blue dot closest to the red dot defines the chosen value of λ.
f) Distribution of positions of detected >1 second pauses relative to the sequence positions of the major sites 'a', 'b', 'c', 'd' and 'his'. Red dashed lines indicates the ±3 bp range.
g) Comparison of pause site crossing time distributions calculated using direct detection and the slowest crossing method on simulated data. For direct detection, crossings that were not detected as pauses were assigned crossing times of 0 seconds, hence the first non-zero crossing time occurs at CCDF < 1.
h) Comparison of pause site crossing time distributions calculated using direct detection (solid lines) and the slowest crossing method (dashed lines) for experimental data collected at 10 pN assisting force. As in panel (g), undetected crossings of the pause sites were assigned a crossing time of 0 s.

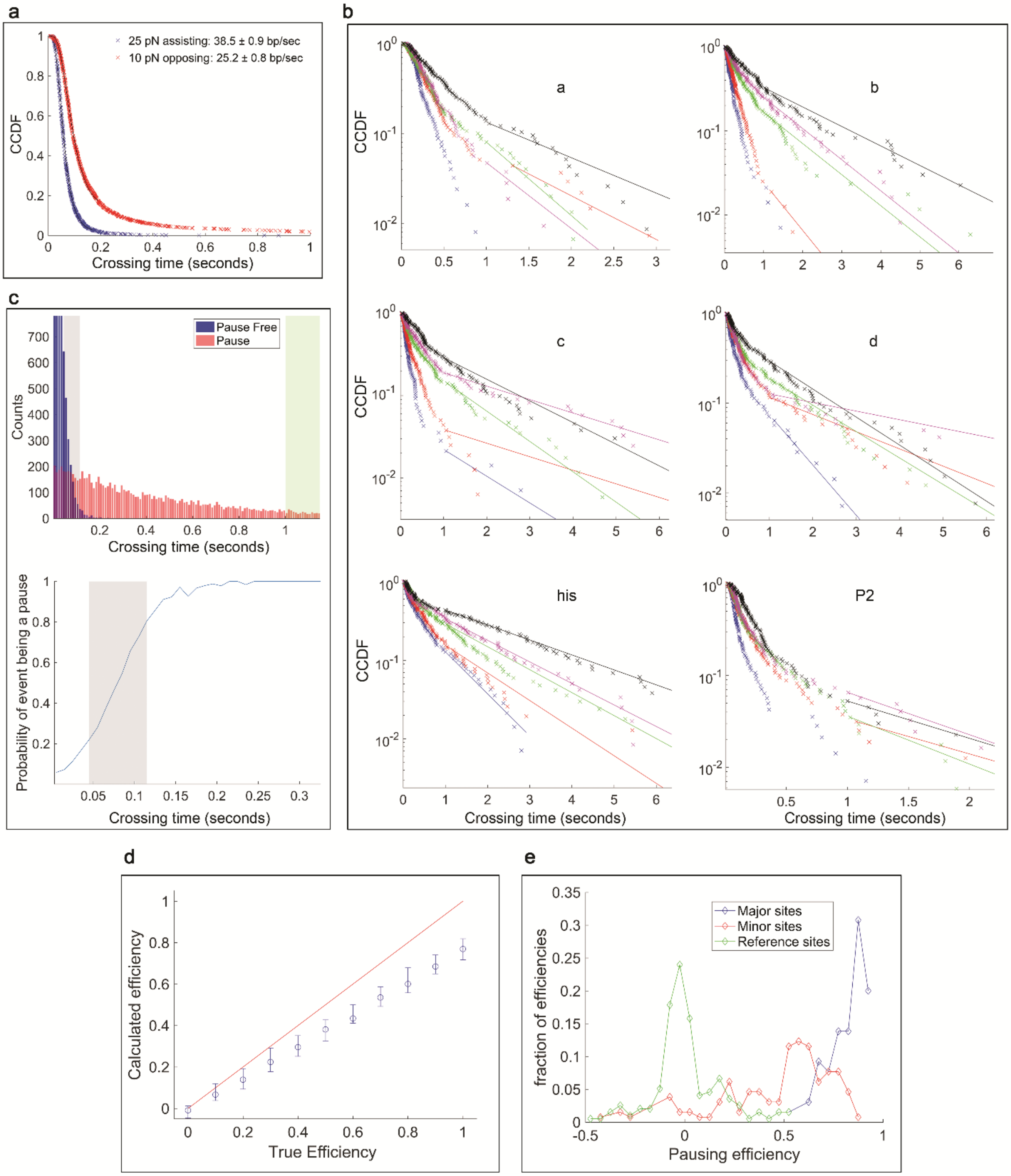

Supplementary figure 2: Analysis of crossing time distributions and pausing efficiency calculations

a) Pause site crossing time distributions calculated at 16 pause free sites and aggregated, at 25 pN assisting force and 10 pN opposing force. The data was fitted to a maximum of six exponentials with the same rate. We fit the data using successively truncated data sets (by removing long events) and selected the fit with the best Kolmogorov-Smirnov statistic when compared to the experimental data (fitting ~98% of the 25 pN assisting force data and 80% of the 10 pN

opposing force data).

b) Exponential fits to crossing times in the 1-20 seconds range for the major pause sites and P2 at five assisting forces. For pauses ‘a’ and P2 at 25 pN, the fit did not converge.

c) (Top) Illustration of the overlap between crossing time distributions for paused states and pause-free states. For each curve, 10,000 points were drawn from exponential distributions with a mean of 0.025 seconds (blue, pause-free), or 0.5 seconds (red, pause). Events longer than 1 second (green shaded area) can only be pauses; events 50-100 ms long have similar probabilities of being derived from paused states or pause-free states (gray shaded area). Exponential distributions were selected for simplicity; the observation would apply regardless of the shape of the distribution of pause crossing times. (Bottom) The probability that an event with a particular duration arises from the paused state was calculated for the case of 50% pausing efficiency (half of the events arise from the paused state). The probability increases with the duration of the event.

d) Calculated efficiency vs. real efficiency in a simulated dataset. Error bars indicate 25-75 percentiles for 100 bootstrapped sets. See supplementary methods for details.

e) Calculated pausing efficiencies at different sites, calculated across the whole data set. Negative estimates of pausing efficiencies correspond to sites (in general, pause-free sites) where the crossing time distribution is faster than the average reference distribution used for comparison.

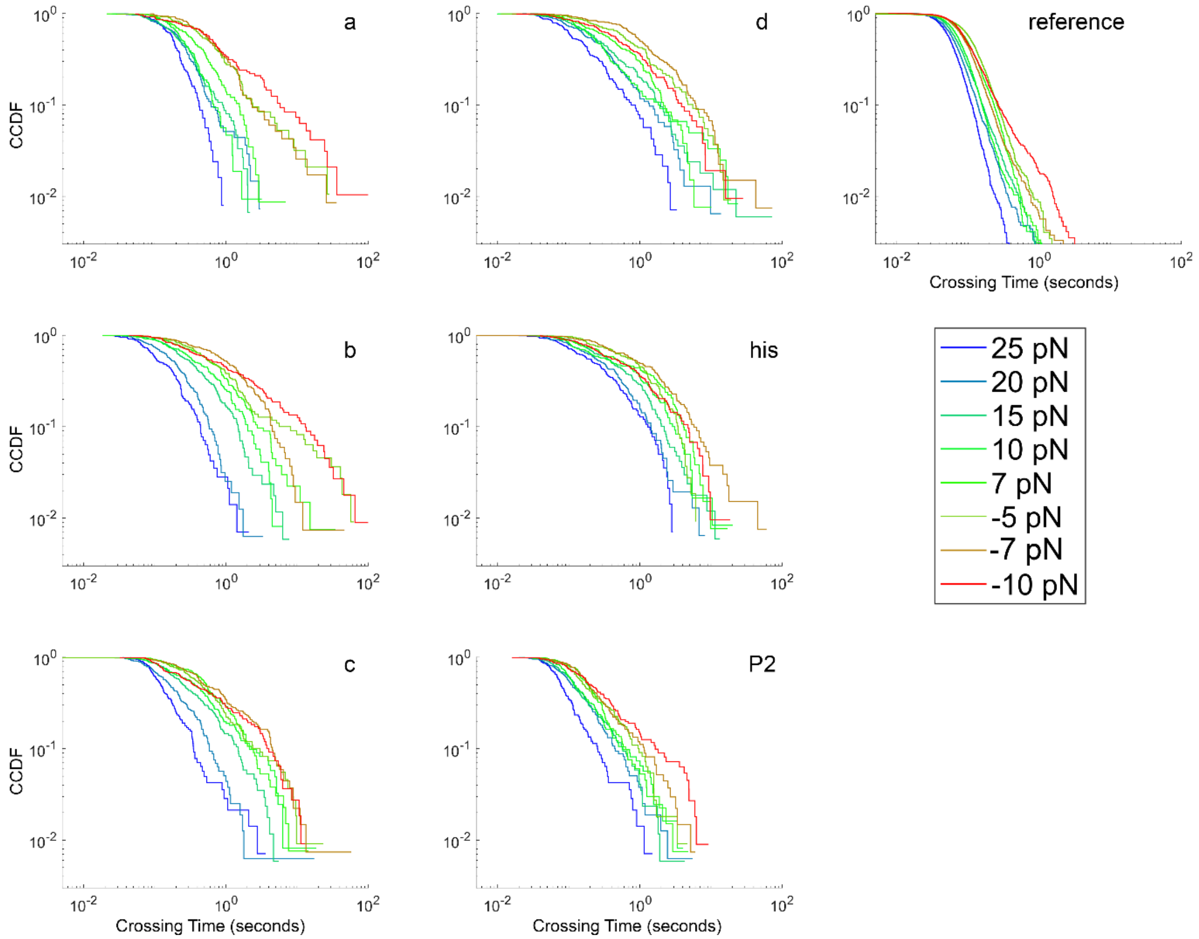

Supplementary figure 3: Pause site crossing time distributions at various pause sites and at reference sites at different applied forces.

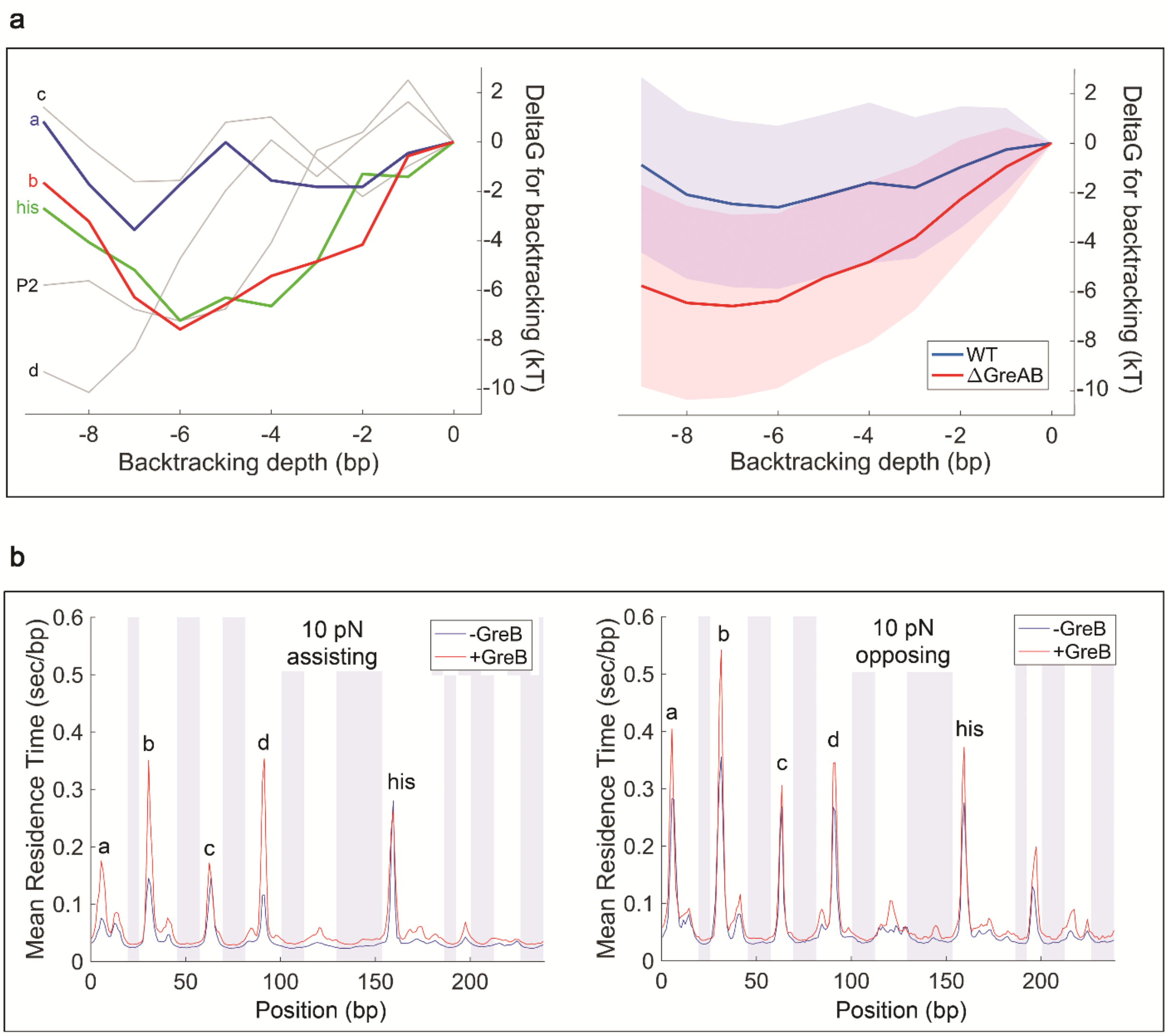

Supplementary figure 4: Thermodynamic driving force for backtracking at different pause sites.
a) (left) Calculated change in free energy of transcription bubble during backtracking at different pause sites. Energies were calculated for a 10 bp RNA:DNA hybrid and a 12 bp transcription bubble. At sites 'a' and 'b' backtracking is energetically favorable to at least 3-4 bp without encountering significant barriers, which is not the case for sites 'P2', 'd' and 'c. energetic gain during backtracking. The 'his' site also shows a large energetic gain, which is most likely offset by the hairpin in the nascent RNA. (Right) Identical calculation averaged across the backtrack-prone sites mapped by Imashimizu et al, for both the WT (blue) and the Δ*greAB* (red) datasets. Solid line, mean difference; shaded area, one standard deviation.
b) Effect of GreB on transcription dynamics at 10 pN assisting force and 10 pN opposing force.

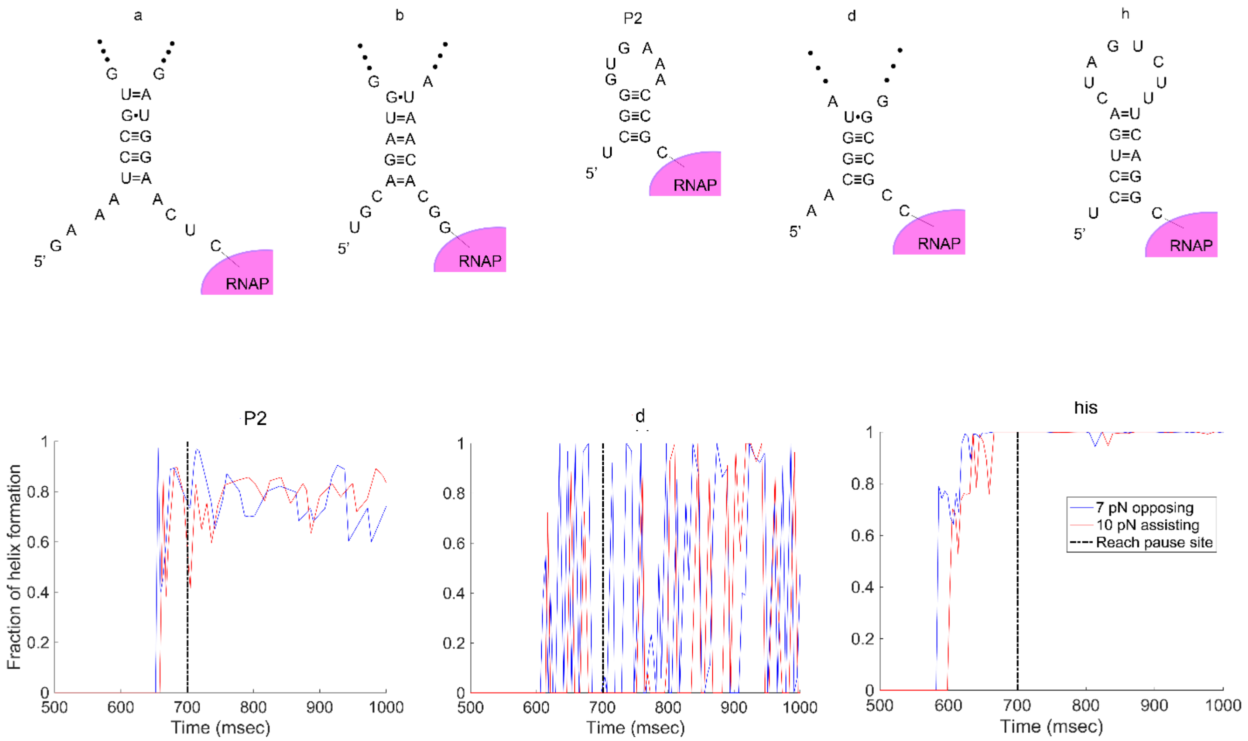

Supplementary figure 5: Kinefold analysis of RNA structures in the pause sites.
(Top) Representative RNA structures formed near the exit channel when RNAP is located at the pause site. (Bottom) Helix traces for the structure shown for sites 'P2', 'd' and 'his'. The black dashed line indicates the completion of transcription of the 50 base RNA, equivalent to the arrival of RNAP at the pause site, thereby releasing the -11 RNA base from the exit channel.

Supplementary Tables:

Supplementary Table 1: Sequences of identified pause sites

Site name	Sequence (-11 to +1)	# consensus bases
a	CGGGTAGATCCG	3/3
b	GGTGAAACCGCA	2/3
c	CGGTAAAGTGTA	2/3
d	CGTATCACTGCG	3/3
his	CGATGTGTGCTG	3/3
P1	TCCGCCCGCATA	1/3
P2	CATCTTTTGACA	1/3
P3	AGGCCGCCGTAT	1/3
P4	ACCACCATCATC	1/3
P5	AAGACATTCAGA	0/3
P6	AGATCGACCTGT	1/3
P7	TTGAAAAAGTTA	1/3
P8	AATCCGTGATAA	0/3
P9	TAACAAGCTGCA	1/3

* Underlined base indicates the 3’ end of the RNA (for sites ‘a’, ‘b’, ‘c’, ‘d’ and ‘his’: known from bulk assays and single molecule data; for sites P1-P9: estimated from single molecule data). Yellow-marked bases indicate bases from the consensus motif $G_{-10}Y_{-1}G_{+1}$.

Supplementary Table 2: pause-free velocities at different conditions

Condition	Velocity (bp/s)
25 pN	42.7 ± 0.3
20 pN	37.6 ± 0.3
15 pN	33.5 ± 0.3
10 pN	34.4 ± 0.2
10 pN + GreB	26.1 ± 0.2
10 pN + RNAse	32.0 ± 0.3
7 pN	28.4 ± 0.3
-5 pN	26.2 ± 0.2
-7 pN	28.3 ± 0.2
-7 pN + GreB	19.4 ± 0.2
-7 pN + RNAse	24.6 ± 0.3
-10 pN	22.9 ± 0.2
-10 pN + GreB	18.9 ± 0.2

* Errors are 95% confidence intervals estimated by bootstrapping.

Supplementary Table 3: Number of traces and analyzed repeats per data set:

Data set	Number of traces	Repeats analyzed (including partial repeats)
25 pN	18	152
20 pN	22	184
15 pN	23	195
10 pN	17	139
10 pN + GreB	16	131
10 pN + RNAse	26	218
7 pN	17	144
-5 pN	17	142
-7 pN	18	153
-7 pN + GreB	17	148
-7 pN + RNAse	19	159
-10 pN	16	119
-10 pN + GreB	17	144

Supplementary Table 4: Measured pausing efficiencies

Force (pN)	‘a’	‘b’	‘c’	‘d’	‘his’	‘P2’
25	0.90-0.94	0.66-0.77	0.84-0.90	0.60-0.70	0.74-0.84	0.33-0.53
20	0.80-0.90	0.65-0.77	0.67-0.76	0.71-0.78	0.75-0.81	0.48-0.58
15	0.83-0.91	0.87-0.93	0.75-0.84	0.82-0.91	0.85-0.91	0.46-0.59
10	0.75-0.91	0.80-0.86	0.77-0.88	0.70-0.78	0.73-0.83	0.42-0.70
7	0.71-0.87	0.85-0.90	0.82-0.93	0.70-0.82	0.66-0.76	0.60-0.70
-5	0.83-0.90	0.82-0.89	0.69-0.86	0.76-0.83	0.86-0.93	0.28-0.51
-7	0.78-0.87	0.87-0.93	0.80-0.87	0.88-0.93	0.84-0.89	0.64-0.75
-10	0.75-0.83	0.74-0.89	0.43-0.6	0.66-0.74	0.73-0.82	0.46-0.57
10 + GreB	0.85-0.92	0.89-0.95	0.83-0.92	0.84-0.90	0.81-0.89	0.48-0.64
-7 + GreB	0.88-0.93	0.87-0.92	0.73-0.87	0.81-0.87	0.68-0.86	0.58-0.67
-10 + GreB	0.82-0.90	0.76-0.84	0.61-0.70	0.77-0.89	0.64-0.76	0.64-0.76
10 + RNase	0.88-0.93	0.74-0.83	0.78-0.86	0.65-0.75	0.50-0.72	0.73-0.82
-7 + RNase	0.84-0.92	0.83-0.89	0.67-0.78	0.75-0.83	0.52-0.71	0.77-0.84

* Positive forces indicate assisting geometry and negative forces indicate opposing geometry. Efficiencies are given as 25th-75th percentile ranges of 100 bootstrapped values.

Supplementary Methods:

Initial selection of data

Prior to data analysis, we inspected the traces for irregularities. First, in a small subset of the traces, the breaking of the tether left a longer tether still connecting the beads, which indicates more than one molecule was tethered between the beads. These traces were discarded from further analysis. In 12 traces, large rips (20-100 nm size) were observed at early stages in the traces, followed by normal transcription and clean breaking at the end. In these traces only data following the rip was used.

Second, during the experiments we determined that photodamage can be a significant effect in such systems, despite the addition of singlet oxygen scavengers. To test this, we performed experiments in which we held the beads in the traps for several minutes before attempting to obtain tethers. Tethers obtained this way were almost always inactive or exhibited extremely slow activity (>3-4 slower than average rates). We attribute this effect to the fact that before a tether is formed, the polymerase is frequently very close to the surface of the bead, where most reactive oxygen species are generated[1], while a tethered polymerase is far away from the surface and is thus better protected. When obtaining tethers <1 minute after trapping the bead, the majority of the tethers exhibited activity and tethers showing exceptionally slow activity typical of photodamage were identified easily and removed from analysis.

The final data set contained 432 transcription traces, out of which 251 traces reached the repeat region without entering a long-lived pause (>200 seconds) or premature breaking of the tether.

Repeat analysis

In theory, since we have exact knowledge of the starting point of each trace (the starvation site), if we had an accurate measurement of the force on the tether and the worm-like chain parameters of the template under the experimental conditions, we would be able to accurately calculate the position of the polymerase on the template throughout the trace from the starting point. These conditions are not met for several reasons:

1. There is a small uncertainty in the starting position, since chasing the polymerase requires moving the beads in the chamber to a different location with a different buffer composition, which as mentioned before introduces small changes to the force (or trap distance under force feedback). Furthermore, the polymerase starts transcribing while the chamber is moving. This mixes the signal coming from transcription with signals coming from the viscous drag on the beads during the motion, which increases the uncertainty in starting position.
2. Bead sizes and viscosity of the buffer are usually not precisely known, and therefore there is an uncertainty in the measured forces. This will change the conversion between nanometers and base pairs. This effect is apparent in our data (Supplementary figure 1) – the spread of the nm length of the 239 base pair repeat around the mean is 0.5-1%. Over a template 3000 base pair long, this may result in an error of 15-30 base pairs in positioning. Moreover, this error will not be distributed evenly across the trace, but will grow as the polymerase transcribes.

An alignment method based on a repeating pattern throughout the template increases the accuracy in the positions of an individual RNAP to ± 3 base pairs (as we will show later), and enables us to study the behavior at specific sites by observing multiple crossings of those sites. We present here the algorithm we used for the alignment of the traces collected on the repeating template.

Initial processing of data

The first step in the alignment procedure is the calculation of length in nm of the 239 bp repeat, which we term the *physical repeat length (PRL).* This number is used to convert the physical distance transcribed by RNAP to the position on the template sequence. Since the traces were collected at a constant force, a constant PRL applies throughout each trace.

The repeats are located between 1107 and 3019 bp downstream of the starvation site. As an initial approximation, starting from an average rise per bp of 0.33 nm, we thus estimated the position of the repeats as the region of the trace located between $1107 \times 0.33 \times 1.02\,\text{nm} = 363\,\text{nm}$ and $3019 \times 0.33 \times 0.98\,\text{nm} = 1005\,\text{nm}$ after the starting position (where the factors of 1.02 and 0.98 are used to avoid including non-repeat region). This approximation will be revisited later.

At $0.33\,\text{nm}/\,\text{bp}$, the average PRL is 79 nm. Our algorithm requires the presence of at least two full repeats in order to align a trace; thus, we discarded any trace which covered less than $2 \times 82\text{ nm} = 164\,\text{nm}$ of the repeats (where we used a slightly larger number for the period to take into account to take into account the initial uncertainty in the period).

Residence time histogram

We started by computing a residence time histogram of the data; that is, we discretized the distance axis in small bins and asked, how much time did the trace spend in each of these windows? As the following discussion will make clear, the size of these bins must be chosen well below the expected size of a base pair; in order to work with round numbers, we set it to $(1/40)\,\text{nm}$, which is approximately $(1/13)\,\text{bp}$. Crucially, we find that, at least for the first step of the analysis (periodization), we do not need to downsample the data before computing the histogram; that is, the dataset at full bandwidth (800 Hz) is used to populate the histogram.

At regions of high transcription rates (as high as 40 bp/s), there will be 20-40 data points per base pair, which is 1-4 data points per bin. At such conditions the occasional empty bins are unavoidable. Such empty bins are problematic for the further analysis. Thus, we instead connected consecutive points in the trace and populated histogram bins in proportion of what fraction of the connecting segments fell within each bin. Such an approach also ensures that the residence time histogram changes continuously when the size or the origin of the bins in changed, rather than by discrete jumps whenever a data point crosses the edge between two bins.

Periodization

Having computed the residence time histogram (RTH) over the (expected) repeat region, we relied on the following observation in order to determine the correct nm-to-base pair conversion factor: the RTH over one repeat should be similar to the RTH over another repeat. Thus, we can try various candidate conversion factors (taken in a small range around the Extensible Worm-Like Chain prediction), corresponding to various values of PRL (for a 239 bp repeat, PRL = 75-81 nm, depending on force) and, for each of these conversion factors, quantify the similarity as suggested above. The PRL with the highest similarity score is then taken as the correct one.

Herbert et al. (2006)[2] noted that such an evaluation of similarity can be carried out by folding the RTH over itself using the given PRL (that is, by summing the values in the RTH at positions $x, x + PRL, \ldots$ for each x). When the period is correctly chosen, the folded RTH should present large spikes due to the summing in phase of the pausing events over each of the repeats; in other words, some positions in the repeat should exhibit very long residence times. Thus, they computed the

distribution of residence times in the folded RTH, and used the skewness (normalized third moment) of the log-residence-time distribution as the similarity score.

We found, instead, that a cross-validation style approach performs better to compute the similarity score. Specifically, we remove one repeat (the *testing set*), compute the folded histogram on the rest of the data (the *training set*), and compute the probability of observing the testing set under the hypothesis that the folded histogram gives the correct distribution. We then repeat this operation using each possible testing set, and use the average log-probability across all testing sets as the scoring criterion. The scheme is described graphically in supplementary figure 1a.

To clarify, we write down the equations here. Let r_i be the residence time at position $i = 0, \ldots, N - 1$ and P be the tentative period. We write $S_a^b = \sum_{i=a}^{b-1} r_i$ (sum of residence times from a to b), $S_{i[P]} = r_i + r_{i+P} + \cdots$ (sum of residence times at positions an integer number of periods from i), $S = \sum_i r_i$ (total residence time).

Consider the testing set corresponding to positions $i, \ldots, i + P - 1$. The observed distribution from the testing set is $f_{i,j} = r_j / S_i^{i+P}$ (histogram counts at position j, divided by the total counts between i and $i + P$). The reference distribution for the training set is $g_{i,j} = (S_{j[P]} - r_j)/(S - S_i^{i+P})$, where $i \leq j < i + P$ (folded counts at position a multiple of P away from j, excluding j itself, divided by total counts, excluding counts between i and $i + P$). The probability of observing the testing set given the training set is

$$L = \prod_{j=1}^{i+P-1} g_{i,j}^{r_j};$$

thus, the log-likelihood for this testing set is thus

$$LL_i = \sum_{j=i}^{i+P-1} f_i\,(j)\log g_i(j) = \frac{1}{S_i^{i+P}} \sum_{j=i}^{i+P-1} r_j \log \frac{S_{j[P]} - r_j}{S - S_i^{i+P}}$$

(after normalization by $1/S_i^{i+P}$.)

We now need to average these likelihood over all possible testing sets, that is, $i = 0, \ldots, N - P - 1$:

$$\begin{aligned} LL &= \frac{1}{N-P} \sum_{i=0}^{N-P-1} LL_i \\ &= \frac{1}{N-P} \sum_{i=0}^{N-P-1} \frac{1}{S_i^{i+P}} \left[\sum_{j=i}^{i+P-1} r_j \log\left(S_{j[P]} - r_j\right) - r_j \log\left(S - S_i^{i+P}\right) \right] \\ &= \frac{1}{N-P} \sum_{i=0}^{N-P-1} \left[\frac{\sum_{j=i}^{i+P-1} r_j \log\left(S_{j[P]} - r_j\right)}{S_i^{i+P}} - \log\left(S - S_i^{i+P}\right) \right], \end{aligned}$$

where we have relied on the fact that $\sum_{j=i}^{i+P-1} r_j = S_i^{i+P}$.

The partial sums S_i^{i+P} and $\sum_{j=i}^{i+P-1} r_j \log\left(S_{j[P]} - r_j\right)$ can be computed efficiently by first computing the cumulative sums starting from index 0 and then taking the difference between the cumulative sums at the two endpoints of the sum.

While this approach works well for finding the correct period size of traces that exhibit moderately strong periodic pausing, we found that for traces where the pausing is very weak (but otherwise collected at the same force), the estimated period size tends to be too small. We believe that this is due to the fact that the computation of similarity scores for different periods (expressed in terms of physical size) compares the RTH over different numbers of bins. For example, consider the case of a perfectly uniform RTH with P bins (each with a relative occupancy of $1/P$). The log-likelihood for any training set would be

$$LL_i = \sum_{j=i}^{i+P-1} \frac{1}{P} \log \frac{1}{P} = -\log P$$

Thus, we need to correct the similarity score by taking $-\log P$ as the base score (which effectively penalizes small periods): the final similarity score that we maximized in order to find the physical period size was $LL + \log P$.

The period sizes are expected to increase with force as the DNA becomes more extended. This is observed when plotting the period sizes versus the force (Supplementary figure 1b). Fitting the mean period sizes obtained at each force to an extensible worm-like chain model yielded a persistence length of 30 ± 18 nm; the stretch modulus could not be fitted with high accuracy (0-10000 pN). The value of the persistence length is consistent with established parameters for DNA.

Inter-trace alignment

Having computed the correct period size, or nanometer-to-base pair conversion factor, for each trace, we next needed to find the relative position offset between each trace (all distances being now expressed in bp). For this purpose, we relied on a similar cross-validation strategy: we shifted the RTH of each trace upstream or downstream until they matched each other as well as possible, as measured by the average likelihood of observing a given RTH (testing set) if the underlying probability distribution is given by the average of the other RTHs (training set).

Note that, in order to align various traces with each other, the bin size of the RTHs need to be identical; a new RTH was thus computed for each trace with a bin size of $(1/10)$ bp (using the same procedure as above). Additionally, we found that for this purpose, a better quality alignment was obtained by downsampling the traces before computing the RTH; the traces were downsampled to 5 Hz.

In this case, the expression for the average log-likelihood is simpler. We define $r_{u,i}$ the average residence time of the u-th trace at position i, $S_u = \sum_j r_{u,i}$ (total average time for trace u), $S_i = \sum_u r_{u,i}$ (total time at position i) (note the abuse of notation), and $S = \sum_{u,i} r_{u,i}$. The log-likelihood associated with the u-th trace is (similarly to above)

$$LL_u = \sum_i r_{u,i} \log \frac{S_j - r_{u,i}}{S - S_u}$$

and the average log-likelihood across training sets is

$$\begin{aligned} LL &= \sum_u LL_u \\ &= \sum_{u,i} r_{u,i} \left[\log\left(S_j - r_{u,i}\right) - \log(S - S_u)\right] \\ &= \sum_{u,i} r_{u,i} \log\left(S_i - r_{u,i}\right) - \sum_u S_u \log(S - S_u). \end{aligned}$$

The second term is independent of the relative offsets between the tethers and can be ignored.

Unfortunately, due to the large number of parameters that need to be optimized simultaneously (one offset for each trace, except the first trace which can be taken as an (arbitrary) reference), and the presence of multiple local maxima, we found that the global optimizer on which we relied (scipy.optimize.basinhopping) is unable to find a satisfactory solution.

We thus relied on a simpler approach first: we computed, for each pair of average residence time histograms $(r_{u,i})_i$, $(r_{v,i})_i$ the correlation between the two traces ($(r_u * r_v)_i = \sum_j r_{u,j}\, r_{v,i+j}$), and found the relative shift $\delta_{u,v}$ that maximized this correlation. A natural idea is then to find shifts δ_u for each individual trace such that $\delta_u \ominus \delta_v = \delta_{u,v}$ for all u, v; where $\ominus$ denotes the difference taken modulo the number of bins. However, this system is overdetermined and has no solution; additionally, the presence of the modulo term makes classic linear algebra techniques (e.g. least squares) not directly applicable.

It remains natural to attempt to find an approximate solution for the system $\delta_u - \delta_v = \delta_{u,v}$. One could, for example, attempt to find the minimizer

$$\underset{(\delta_u)}{\operatorname{argmin}} \sum_{u,v} \left(\delta_u \ominus \delta_v - \delta_{u,v}\right)^2$$

using a global optimizer. However, as it turns out, not all $\delta_{u,v}$ are reliable: for some pairs of traces, the maximum in the correlation incorrectly matches two unrelated peaks. Thus, we need to use a robust alternative to least squares, which is able to ignore an unsatisfiable equation as long as most others are properly handled. A classical example is least absolute deviations,

$$\underset{(\delta_u)}{\operatorname{argmin}} \sum_{u,v} \left|\delta_u \ominus \delta_v - \delta_{u,v}\right|$$

An additional correction is needed to make the minimization problem better behaved. A problem of the least absolute deviations formulation is that it is not strictly convex, even locally; specifically, if, say, $\delta_u \ominus \delta_v < \delta_{u,v}$ and $\delta_u \ominus \delta_w > \delta_{u,w}$, then the effect of slightly changing δ_u on these two terms will cancel out each other; that is, the target function is degenerate. We fix this issue by rendering the target function locally strictly convex,

$$\underset{(\delta_u)}{\operatorname{argmin}} \sum_{u,v} \sqrt{\left(\delta_u \ominus \delta_v - \delta_{u,v}\right)^2 + \epsilon^2}$$

where ϵ is a small regularizer, chosen to be equal to one basepair.

It turns out that this target function is well-behaved enough so that a generic global optimizer (scipy.optimize.basinhopping) can find a reasonable minimum for it quickly. This minimum is then

used as an initial point to the log-likelihood maximization problem we formulated in the first place.

Having found the set of offsets that maximize this likelihood, each trace may still be away from their correct position by an integer number of periods. This error is fixed by shifting each trace by an integer number of repeats so that their start position (which, as explained above, is relatively poorly defined due to the need to move from the buffer channel to the NTP channel) is no more than half a repeat away from the expected initial tether length.

In an initial alignment, we set the positions of the major pauses ‘a’, ‘b’, ‘c’, ‘d’, and ‘his’ within the 239 bp repeat to be 5, 30, 63, 90 and 158, respectively, thus maintaining the distance between the pause sites in the sequence. We shifted the complete, aligned residence time histogram to maximize the time spent at those sites (Supplementary figure 1c). First, we observed that site ‘c’ was clearly shifted ~1 bp upstream relative to the expected position. To validate the positions of the pause ‘c’, we performed a bulk transcription experiment covering the region of pauses ‘c’ and ‘b’ (supplementary figure 1d). The results show a strong pause site 1 bp upstream compared to the previously known position of pause ‘c’, but with a weaker pause following it. The source of the discrepancy is thus unclear. Regardless, we moved the position of pause ‘c’ accordingly. It should be also noted that the observed distance between those sites was not an integer number of base pairs. This may be due to several reasons: a possible difference in translocation state between the pause sites, a difference in the rise per base pair along the template[10], as well as inherent limitations on the accuracy of our data and alignment. We used the detected pause positions at this stage as the expected positions for pause scoring described later.

Remember that at the beginning of our alignment procedure, we needed to assume what section of the trace corresponded to the repeat region, based on an approximate estimate of the size of a base-pair and of the position of the starvation site. As the output of our alignment procedure consists of more accurate estimates of both quantities, we used these outputs to better estimate the position of the repeat region, and repeat the entire alignment procedure (only for one iteration).

Trace regularization

While linear filtering of the data is commonly used as a preprocessing step to detect pause events in an optical tweezers trace, such a method is known to exhibit low sensitivity to fast, sub-second pausing events. Instead, we relied on total variation regularization to compute pause lifetimes[3]. A side-product of this regularization is the ability to detect rips and zips.

Let us consider a trace $y = (y_0, y_1, \dots)$. For traces where no backtracking occurs, a natural way to regularize the trace is *isotonic regression*; namely, finding the trace $\hat{y}$ that is non-decreasing ($y_i \le y_{i+1}$ for all i) and that minimizes the sum of squared errors, $\sum_i (y_i - \hat{y}_i)^2$.

However, backtracking is clearly observed, if somewhat rarely, in our dataset. In order to allow our fit to go backwards whenever needed, we write a target function that penalizes both deviation from the measurements (sum of squared errors) and excessive influence of the noise spikes:

$$\underset{\hat{y}}{\operatorname{argmin}} \left[\sum_i (y_i - \hat{y}_i)^2 + \lambda \sum_i |\hat{y}_{i+1} - \hat{y}_i| \right].$$

For example, note that the second term is equal to the end to end distance of $\hat{y}$ if $\hat{y}$ never moves backwards; each backtrack increases the term by twice the backtrack depth. The factor λ indicates the relative importance we give to the two terms.

Efficient algorithms exist to find the minimizer $\hat{y}$; we relied on the implementation of Johnson's dynamic programming[4] in the prox_tv package.

The remaining question is the choice of the relative weight, λ, between the fidelity term (mean square error) $\sum_i (y_i - \hat{y}_i)^2$ and the regularization term (total variation) $\sum_i |\hat{y}_{i+1} - \hat{y}_i|$. We relied on the L-curve method to pick such a value[5]. The L-curve is the parametric curve obtained by plotting the points $\left(\left(\sum_i (y_i - \hat{y}_i)^2\right)^{1/2}, \sum_i |\hat{y}_{i+1} - \hat{y}_i| \right)$ for various values of λ in log-log scale (we plot the *root* mean square error so that both terms have the dimension of a distance). When λ is small, increases in λ tend to greatly decrease (i.e., improve) the regularization term (by avoiding to follow the large number of fast spikes due to noise), while only minimally increasing (i.e., worsen) the fidelity term (as the smoothed out peaks are small in amplitude). Conversely, when λ is large, increases in λ tend to only minimally decrease the regularization term (because most of the spikes have been smoothed out), while greatly increasing the fidelity term (because all that is left to do is to smooth out the large, true movement in the trace). The presence of these two regimes gives the L-curve an "L" shape, where the two branches correspond to the two regimes described (Supplementary figure 1e). The corner of the L-curve corresponds to a choice of λ where the compromise between the fidelity term and regularization term is, in a sense, optimal.

The end result of this procedure is a trace that consists of exactly flat regions, separated by sharp jumps (this phenomemon is known as the "staircasing effect"[3]) (Figure 1C). Jumps of a size greater than 4 nm were considered as rips or zips in the raw data; whenever one was detected, the trace was split into two sections (before and after the jump) that were analyzed separately.

Calculation of pause site crossing times

The regularized trace was then used for scoring the pauses. Again, remember that the result of the regularization is a trace that is nearly non-decreasing, with backtracks occurring only at a few, discrete positions.

We first converted the trace to a fully non-decreasing one by replacing its value during any backtrack by the maximum value attained so far. Physically, this operation can be understood as tracking the nature of the 3' end of the RNA, rather than the position of the polymerase itself.

We could then compute the pause site crossing time as follows: for each site, we defined a fixed range surrounding the pause site symmetrically, with the range wide enough to ensure that the pause site will be located within the tested range, but narrow enough to exclude other pause sites. Within this range, the polymerase takes steps through multiple pause-free sites and a single pause site. To calculate the crossing time of the pause site, we assumed that the crossing of the pause site was the slowest step, and therefore picked, among all the 1 bp-wide windows that one can draw within the tested range, the window that took the longest to cross (Figure 1c).

For this purpose, we first needed to assess how large this range needs to be to capture most of the pausing events occurring at any site, which is, essentially, a measure of the accuracy of the positioning individual RNAP molecules. The P1 site is located ~8 bp downstream of 'a', and P2 is located ~10 bp downstream of 'b', and they are very clearly resolved. Therefore we set the range of 5 bp on each side as maximum range necessary. To find if we can reduce this range, we applied the pause detection algorithm used previously[2,6,7] with slight modifications. The data was downsampled to 200 Hz and filtered with a 3rd order Savitsky-Golay filter with a 1 second window. The instantaneous velocity was computed at every point and a velocity histogram was calculated using 0.2 bp/sec bins for all the traces collected at each condition. The region from -2.5 bp/sec to

2.5 bp/sec was then fit to a Gaussian. We assumed that all rates lower than the center of the Gaussian are associated with pausing events and that the distribution of rates for pause sites is symmetric. Using this assumption we calculated the histogram for rates associated with pausing above the center. This enabled us to calculate, for every possible rate, what is the probability that this rate is associated with a pause. We defined a threshold rate as the rate below which this probability is at least 90%. We used this threshold to score pauses, while combining any adjacent pauses that were spaced <1 bp apart.

After pause scoring, for each scored pause we found the closest major site. Then, for the ±5 bp range around every major site we plotted a histogram of the distance of scored pauses from the site (Supplementary figure 1f). We found that a range of 3 bp at every direction contains ~94% of the observed pausing events. We therefore chose a range of ± 3 bp for pause scoring.

In order to assess the accuracy of our method, we also generated simulated data corresponding to the crossing of a single pause, as follows. We first generated exponentially distributed dwell times, with a mean of 50 ms, corresponding to dwells at pause-free sites; we generated such steps until their total duration exceeded 2 s (twice the size of Savitsky-Golay filter window). We then generated another dwell time, corresponding to the crossing of the pause site. That dwell time was drawn from an exponential distribution with a longer dwell time, e.g. 500 ms. After the crossing of the pause, we further generated 2 s worth of pause-free dwell times, again with a mean of 50 ms. An optical tweezers trace was constructed by adding the stepping motion corresponding to these dwell times with noise directly taken from long pauses observed in experimental data at 10 pN assisting force (in order to fully match the spectral characteristics of that noise).

We repeated this procedure in order to generate 200 traces. We then analyzed the resultant traces and calculated the pause site crossing times using the direct detection method and the Slowest Crossing method that we developed (Supplementary figure 1g). We also compared crossing time distributions obtained from experimental data using the two methods (Supplementary figure 1h). Both methods showed similar trends for pauses longer than 1 second and sometimes even shorter pauses. However, our new scoring method finds a crossing time for every crossing of the pause site, therefore enabling us to characterize the behavior at very short time scales.

Analysis of crossing time distributions

When performing this calculation, we essentially measure the slowest step between six steps taking place in the analyzed window (given our localization accuracy of ± 3 bp). In pause-free regions, this will naturally result in crossing times that are longer than the average pause-free dwell. Crossing time distributions in pause-free sites can be approximated by a distribution of the maximum of six identical exponentials, yielding transcription rates close to the measured average values (Supplementary figure 2a). The behavior at long time scales at opposing forces is not well fit by this model, possibly reflecting the fact that pausing events that occur outside the pause regions are more frequent under opposing forces than under assisting forces.

At pause sites, the behavior of the calculated crossing times is more complex. If the polymerase happened to have crossed the pause site relatively quickly (for example, because it did not pause or because RNAP was in the paused state for a relatively short time), there is a significant probability that the event captured by our method will be the crossing of one of the pause-free sites surrounding the pause site, and therefore some crossing times measured at short time scales will come from crossings of pause-free sites. As the pause site crossing becomes longer, the probability of capturing

increases. To estimate the probability of capturing a crossing of the pause site, we assumed the five surrounding pause-free steps are exponentially distributed and calculated the probability that a pause-site crossing of a given length will be longer than the other five steps. The calculation shows that pause-site crossings as short as 4.5 times longer than the average pause-free dwell (equivalent to 125-250 ms) will be captured with >95% probability. A significant fraction of shorter (50–100 ms) crossings will also be captured. Indeed, as shown in the main text, the crossing time distribution at pause sites can be distinguished from the distribution at pause-free sites even at very short (<200 ms) time scales.

Estimation of pausing efficiencies using the non-parametric method

Previous studies[2,6] have computed pausing efficiencies by fitting the tail of the distribution of pause lifetimes to a single exponential distribution, and interpreting the difference between the number of fast events estimated by the fit and the actual number of observed fast crossings as the non-paused fraction. To test this method, we tried to fit the 1 – 20 second region in the crossing times to an exponential distribution (Supplementary figure 2b). We encountered several issues: first, an exponential distribution did not give good fits consistently for any site. Second, in most sites the crossing time log-cumulative distribution becomes steeper at the 0.5-1 second time scale compared to longer time scales, indicating faster dynamics dominating at this time scale; that is, there are more 0.5-1 second events than would be expected from an exponential extrapolation from longer time scales. In other words, extrapolation to t=0 from events longer than 1 second inherently underestimates the pausing efficiency, with the effect probably being the greatest at high assisting forces, where very few of the events are longer than 1 second. Finally, at weak pause sites, or at high assisting forces, the number of events longer than 0.5-1 sec was frequently very low, making such fits very unreliable. We attempted instead to provide a fully non-parametric approach to the evaluation of pausing efficiency, inspired from the analysis of power law tails by Clauset et al[8]. This approach utilizes the full crossing time distributions at the pause sites. We assume that for a certain fraction of the observed crossing times, RNAP did not enter a paused state, and that the crossing times of these pause-free events have the same distribution as the crossing times measured at the pause-free sites (the *reference* distribution). The remaining events arise from the paused state, and their fraction is the pausing efficiency. As explained in the main text, long events arise from the paused state with very high probability, while events in the 50-150 msec range have similar probabilities of arising from a pause or from pause-free dynamics (Supplementary figure 2c). The probability that a particular event arises from a pause increases gradually as the crossing time increases.

Let us consider a distribution of crossing times at the pause sites, and a distribution of crossing times at the reference sites. Imagine that we were only able to observe, both at pause and at reference sites, the events that lasted no longer than a cutoff time τ (and were oblivious even to the existence of longer events). We could test how well the pause and reference distributions match each other by computing a distance statistic between the distributions (in our case, the Kolmogorov-Smirnov statistic). We compute this statistic for all possible τs, and pick the one for which the two distributions are the closest to each other as the cutoff time (typically, if τ is chosen too small, then statistical fluctuations due to small sample size lead to a larger distance between the truncated distributions; if τ is chosen too large, it is the intrinsic difference between the underlying distributions that leads to a large distance between the truncated distributions) (figure 2A).

While it is clear that at values of τ higher than the cutoff the crossing time distribution at the pause

site is clearly different from non-pause sites, it is less clear what happens below the cutoff. At the most extreme scenario, all events under the cutoff are non-paused (that is, are derived from the same distribution as in pause-free sites). If we make this assumption, and also assume that the subset of events in the pause site that are pause-free have the same distribution as observed in pause-free sites, we can estimate from the reference distribution, what additional number of events *longer* than the cutoff we would observe at a non-pause site; we add this number to our estimated number of non-pause events at the pause site. The ratio between the remaining events at the pause site (which we considered to be true pauses) and the total number of events at that site is an estimate of the pausing efficiency – what fraction of the crossing times are derived from a distribution distinct from pause-free behavior (Figure 2A).

However, this estimate for the efficiency is only a *lower bound* for the true pausing efficiency. There are several factor contributing to this:

1. As shown in Supplementary figure 1e, up to 6% of the pauses occurring at every site will not occur in the ±3 bp window around the expected position, and therefore the event most likely to be scored is a pause free event. We can estimate the *maximum* error caused by this effect by making the following assumptions: first, that 6% of events in the crossing time distribution have no pause site in the window, and that all of these events are recorded as pause free; second, that the missed pause site crossings have the same pausing efficiency as the captured crossings. Under those assumptions, the true pausing efficiency E is

$$E = \frac{N_{\mathrm{p,in}} + N_{\mathrm{p,out}}}{N_{\mathrm{tot}}}$$

where N_{tot} is the total number of crossings, $N_{\mathrm{p,in}}$ the number of paused crossings that occurred in the window, and $N_{\mathrm{p,out}}$ the number of paused crossings that occurred out of the window – the latter being equal to $N_{\mathrm{tot}} \times 0.06E$.

Meanwhile, the observed efficiency is only derived from paused crossings in the window, i.e.

$$E_{\mathrm{obs}} = \frac{N_{\mathrm{p,in}}}{N_{\mathrm{tot}}} = (1 - 0.06)E = 0.94E$$

Therefore, an observed efficiency of 80% will correspond to an efficiency of ~85%. However, it should be noted that the fraction of pauses occurring outside the window may be lower than 6% as explained above, and there this effect may be smaller.

2. Our observed cutoff times are low, and at these time scales even datasets derived from completely different distributions (equivalent to 100% pausing efficiency) may not always be distinguishable, particularly with the size of our data sets (150-200 events for pause sites, ~2000 events for non-pause sites).

3. As shown in our simulated data, the slowest crossing method slightly overestimates the true crossing time, by roughly a pause-free dwell time. This overestimation is independent of the true crossing time, and therefore in relative terms, crossings of pause-free sites are overestimated more than crossings of pause sites. Hence the true difference between the CTD at pause sites and the reference CTD (calculated at non-pause sites) is in fact greater than what we measure. This leads to further underestimation of the pausing efficiency.

To estimate the degree of underestimation, we again simulated transcription data through pause sites. In this case, we varied the true pausing efficiency (the fraction of traces in which the crossing time of the pause site is drawn from a slow distribution, and not from the same distribution as pause-free sites). We generated data sets with similar size to the experimental data sets – 200 crossings of the pause sites (at varying efficiencies) and 2000 crossings of non-pause sites (for the reference distributions). Crossing times of non-pause sites were modeled as exponential with a mean crossing time of 50 ms, and crossing times of pause sites were also modeled as exponential with slower means. We found that in such a case, the calculated pausing efficiencies from our method significantly underestimate the true efficiency. Supplementary figure 2d shows that for a mean pause duration of 0.5 s, calculated pausing efficiencies of 70-85% correspond to true efficiencies ~20% higher. The effect is smaller for longer mean pause durations but is still significant at 10-15%.

As a control we measured the efficiency of individual reference sites against all the other reference sites. As expected, reference sites showed pausing efficiencies around 0 (including some negative estimates, corresponding to the case where events at a specific reference sites tend to be *faster* than the global reference distribution), and much lower than both major and minor pause sites (Supplementary figure 2e).

Analysis of the thermodynamic driving force for backtracking

We tested whether the sequence-dependence of backtracking could be explained by the changes in the thermodynamic stability of the transcription bubble as RNAP backtracks. For every position along the template, we calculated the free energy change of forming of the transcription bubble, involving the melting of 12 bp of DNA hybrid (from the -12 position to the active site) and the formation of a 10 bp RNA-DNA hybrid, using tabulated nearest-neighbor free energy values[9,10]. We then calculated the free energy change associated with backtracking by up to 8 bp at each of the pause sites (supplementary figure 3a, left). Only sites 'a', 'b' and 'his' exhibited an energetic gain from backtracking without the need to cross a significant energetic barrier. This observation suggests that a significant part of the large backtracking propensity at sites 'a' and 'b' may be attributed to the gain in free energy resulting from moving the transcription bubble backwards. Although backtracking at the 'his' site appears energetically favorable in this simplified model, it is most likely inhibited by the hairpin in the nascent RNA. More generally, additional sequence-dependent factors that may affect the backtracking propensity are other secondary structures in the nascent RNA[11] and interactions between the nucleic acids and the polymerase itself.

To test whether bubble thermodynamics contribute to backtracking propensity in the bacterial cell, we analyzed the pause sites mapped by Imashimizu et al[12]. We used the same technique to calculate the bubble free energies at the pause sites (supplementary figure 3a, right). At the sites mapped for WT *E. coli*, each of the first three steps were energetically favorable at the majority (at least 55%) of the sites ($p<0.02$, two-sided binomial test) with the least favorable step (the first one) averaging 0.16 kcal/mol across all sites. At the sites mapped for *ΔgreAB E. coli*, each of the first seven steps were energetically favorable at the majority (at least 58%) of the sites ($p<0.01$, two-sided binomial test) with the least favorable step (the seventh) averaging 0.14 kcal/mol across all sites. Conversely, we found that across all annotated transcribed sequences in the E. coli genome (NC_007779.1), sites where backtracking is energetically favorable are in a slight minority (49.95%, $p<0.05$, two-sided binomial test). Overall, these findings support a model in which the differences in the

energetic cost of opening a bubble at various sites are a major contributor to the difference in backtracking propensity. This result is consistent with recent findings from genome-wide analysis of pausing in bacteria and yeast[13].

Kinefold analysis

For the pause sites ‘a’, ‘b’, ‘c’, ‘d’, ‘his’, and P02, we simulated the cotranscriptional folding of 50 RNA bases with the 3’ end at the -11 position relative to the pause site (immediately upstream of the exit channel). We performed the simulation using two transcription rates: 29.1 msec/base (rate at 10 pN assisting force) and 35.3 ms/base (rate at 7 pN opposing force). For every simulation, we checked whether among the five most stable structures there is a structure containing a helix that ends immediately upstream of the polymerase at the pause site (positions -15 to -11). We plotted the helix trace for such helices (% of formation of the helix as a function of time). The results are shown in supplementary figure 4. For the sites ‘P2’ and ‘his’ a stable helix is formed at least 40 msec before RNAP reaches the pause site, with for site ‘d’ a less stable structure appears ~80 msec before RNAP reaches the pause site. The applied force, through its effect on the transcription rate, does not cause any significant changes to the stability of the formed structural or to the timing of their formation relative to the arrival of RNAP at the pause site.

References for Supplementary Material:

1. Landry, M. P., McCall, P. M., Qi, Z. & Chemla, Y. R. Characterization of Photoactivated Singlet Oxygen Damage in Single-Molecule Optical Trap Experiments. *Biophys. J.* **97,** 2128–2136 (2009).

2. Herbert, K. M. *et al.* Sequence-Resolved Detection of Pausing by Single RNA Polymerase Molecules. *Cell* **125,** 1083–1094 (2006).

3. Chan, T., Esedoglu, S., Park, F. & Yip, A. in *Handbook of Mathematical Models in Computer Vision* (eds. Paragios, N., Chen, Y. & Faugeras, O.) 17–31 (Springer US, 2006). doi:10.1007/0-387-28831-7_2

4. Johnson, N. A. A Dynamic Programming Algorithm for the Fused Lasso and L 0-Segmentation. *J. Comput. Graph. Stat.* **22,** 246–260 (2013).

5. Hansen, P. Analysis of Discrete Ill-Posed Problems by Means of the L-Curve. *SIAM Rev.* **34,** 561–580 (1992).

6. Neuman, K. C., Abbondanzieri, E. A., Landick, R., Gelles, J. & Block, S. M. Ubiquitous Transcriptional Pausing Is Independent of RNA Polymerase Backtracking. *Cell* **115,** 437–447 (2003).

7. Galburt, E. A. *et al.* Backtracking determines the force sensitivity of RNAP II in a factor-dependent manner. *Nature* **446,** 820–823 (2007).

8. Clauset, A., Shalizi, C. & Newman, M. Power-Law Distributions in Empirical Data. *SIAM Rev.* **51,** 661–703 (2009).

9. Sugimoto, N. *et al.* Thermodynamic Parameters To Predict Stability of RNA/DNA Hybrid Duplexes. *Biochemistry* **34,** 11211–11216 (1995).

10. SantaLucia, J. A unified view of polymer, dumbbell, and oligonucleotide DNA nearest-neighbor thermodynamics. *Proc. Natl. Acad. Sci. U. S. A.* **95,** 1460–1465 (1998).

11. Zamft, B., Bintu, L., Ishibashi, T. & Bustamante, C. Nascent RNA structure modulates the transcriptional dynamics of RNA polymerases. *Proc. Natl. Acad. Sci.* **109,** 8948–8953 (2012).

12. Imashimizu, M. *et al.* Visualizing translocation dynamics and nascent transcript errors in paused RNA polymerases in vivo. *Genome Biol.* **16,** 98 (2015).

13. Lukačišin, M., Landon, M. & Jajoo, R. Sequence-specific thermodynamic properties of nucleic acids influence both transcriptional pausing and backtracking in yeast. *PLoS One* **12,** e0174066 (2017).
